# Experimental and Numerical Investigation of Prepreg-RTM Co-Curing Molding Composite Bolted T-Joint under Bending Load

**DOI:** 10.3390/polym16071018

**Published:** 2024-04-08

**Authors:** Tao Zhang, Zhitao Luo, Kenan Li, Xiaoquan Cheng

**Affiliations:** 1 School of Materials Science and Engineering, Beihang University, Beijing 100191, China; 13911997771@139.com; 2 Research Institute of Aerospace Special Materials and Processing Technology, Beijing 100074, China; 3 School of Aeronautic Science and Engineering, Beihang University, Beijing 100191, China; luo0327@buaa.edu.cn (Z.L.); kenan_li@buaa.edu.cn (K.L.)

**Keywords:** polymer composites, T-joint, prepreg-RTM co-curing molding, bending performance, finite element analysis, failure analysis

## Abstract

A set of polymer composite bolted T-joints with a novel configuration consisting of an internal skeleton and external skin was fabricated using a prepreg-RTM co-curing molding process. Experiments were conducted to study their mechanical properties under a bending load. A finite element model with a polymer resin area between the skin and skeleton was established and verified by the experimental results. Then, the damage propagation process and failure mechanism of the joint and the influence of three factors related to the layer characteristics of the skin and skeleton were investigated by the validated models. The results show that the bending stiffness and the yield limit load of the novel composite T-joint are 0.81 times and 1.65 times that of the 2A12 aluminum T-joint, respectively, while at only 55.4% of its weight. The damage of the joint is initiated within the resin area and leads to the degradation of the joint’s bending performance. The preferred stacking sequence of the skeleton is [0/+45/90/−45]_ns_ when primarily subjected to bending loads. The decrease in the bending performance is within 5% of the inclining angle of the skeleton, less than 12 degrees. The more 90° layers in the skin, the better the bending performance of the joints, while the more 0° layers, the poorer the bending performance.

## 1. Introduction

In modern aerospace industry, polymer composites are gradually taking the place of metals in primary aircraft structures due to their high specific strength and high specific modulus [1,2], e.g., a variety of metal joints are being replaced by composite joints. Among these joints, T-joints are widely used for connecting components at the vertical position in wings and tails and have become a hot research topic in the field of composite joint design [3,4].

In recent years, many scholars have investigated the bending performance of composite T-joints of various types and those made through various manufacturing processes [5,6,7,8,9,10,11,12,13]. A comparison of these studies reveals that the predominant form of connection for T-joints is adhesive bonding. Adhesive bonding can significantly reduce the number of components and simplify the assembly process, but the bending performance of the joint mainly depends on the bonding quality and the material properties of the adhesive. In contrast, bolted connections can provide higher reliability and a better bending performance and remain a preferred option for primary load-carrying structures [3]. For instance, wings and tails can be connected to the fuselage via bolted T-joints. In these cases, T-joints with bolted connections need to withstand concentrated bending loads derived from the wing surface. Therefore, further research is needed on the bending performance of bolted composite T-joints.

**Figure 1:**
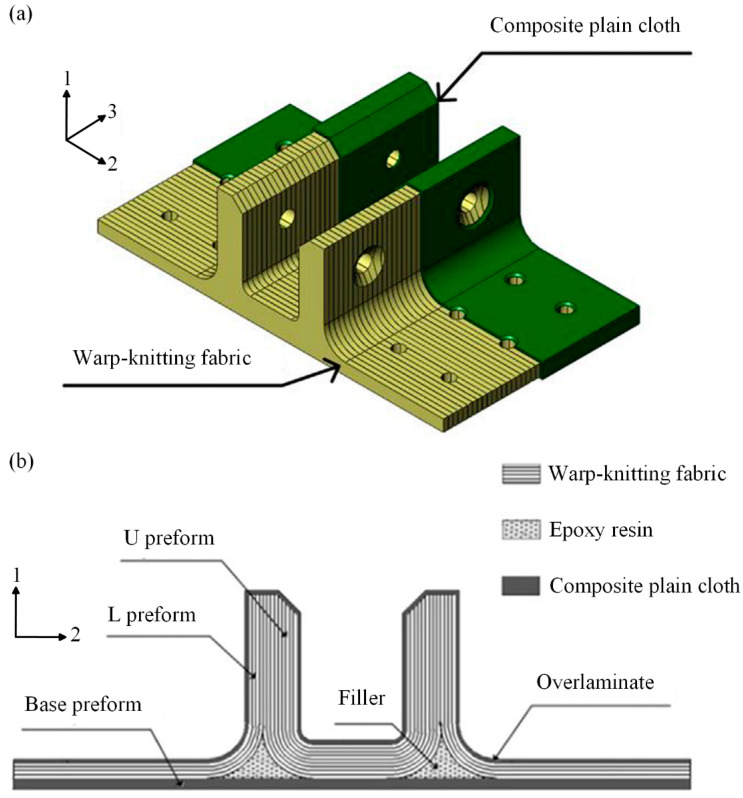
Configuration of bolted π-joint: novel configuration (**a**) and conventional configuration (**b**).

Some studies have found that under bending loads, the primary failure mode of composite T-joints is interlaminar delamination at the R-corner area [10,11,12,13]. Cheng et al. [14,15] investigated the bending performance of a novel configuration composite π-joint. As shown in Figure 1, compared to the conventional π-joint [16], this novel configuration lays the warp-knitted fabric along direction 3, which converts the interlaminar load of the plies at the R-corner area into an in-plane load. Experimental results indicate that compared to a conventional joint of the same size, the ultimate bending load of the novel configuration joint increases by nearly two-fold. It can be seen that converting the interlaminar load into an in-plane load can significantly enhance the load-carrying capacity of composite joints.

Additionally, the manufacturing process also has a significant impact on the mechanical performance of the joints. Some studies have shown that compared to polymer composites based on prepreg molding, products produced by RTM processes have higher fiber volume fractions and higher manufacturing precision. However, RTM preforms are prone to fiber bending deformation and the presence of more voids in the molded products [17,18,19,20]. Li et al. [18] prepared composite laminates fabricated by the prepreg-RTM co-curing molding process and the prepreg compression molding process, respectively. The experimental results indicate that the prepreg-RTM co-curing molding process can effectively reduce the probability of defects such as voids. The interlaminar shear performance and post-impact compression performance of composite laminates fabricated through the prepreg-RTM process are superior to those fabricated using the prepreg compression molding process. It can be seen that if prepregs with high fiber alignment are used in the RTM process, the mechanical performance of RTM products can be effectively improved.

**Figure 2:**
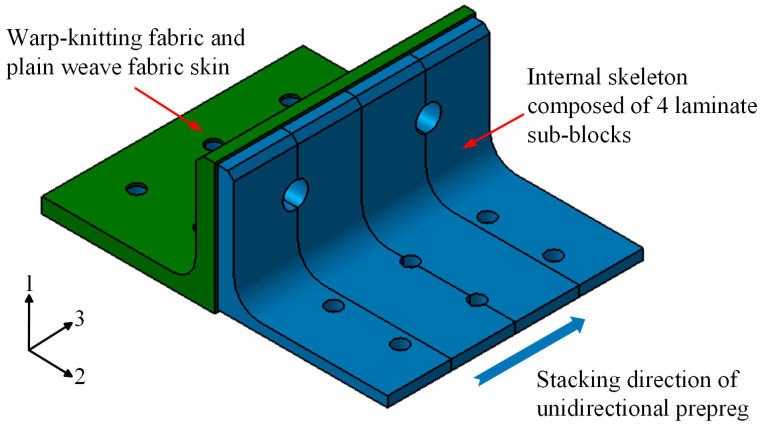
Novel configuration of polymer composite bolted T-joint.

In this present investigation, a set of polymer composite bolted T-joints with a novel configuration consisting of an internal skeleton, in which plies were laid along direction 3, and an external skin was fabricated based on the stacking method of the π-joint with a novel configuration, as shown in Figure 2. Also, the prepreg-RTM co-curing molding process was employed to manufacture the novel T-joint. Then, experimental and numerical investigations were conducted to investigate the mechanical properties and failure mechanism of these joints under a bending load. Finally, based on a validated finite element model, numerical investigations were performed to examine the effects of the stacking sequence and layer inclining of the internal laminate skeleton, as well as the stacking sequence of the skin, on the bending performance of the novel composite bolted T-joint. The achievements of this study can provide references for the high-load-carrying design and preparation strategy of polymer composite bolted T-joints primarily subjected to out-of-plane bending loads.

## 2. Experiment

### 2.1. Specimen Design

**Figure 3:**
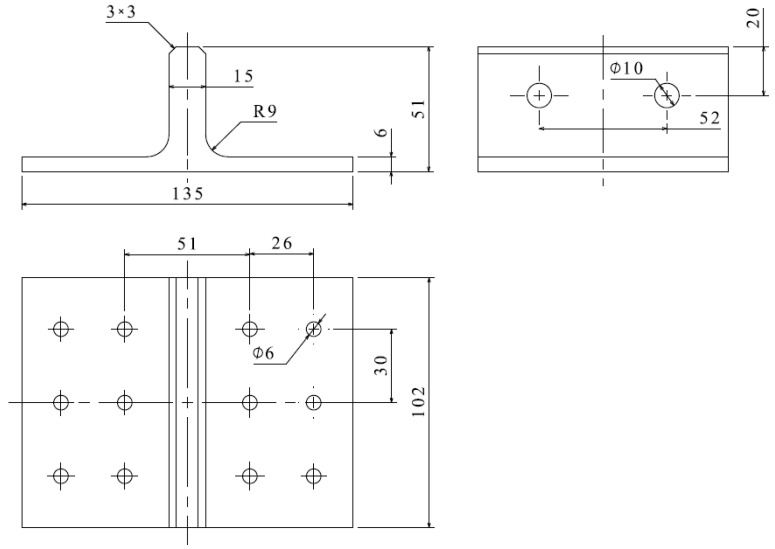
The dimensions of specimen.

**Figure 4:**
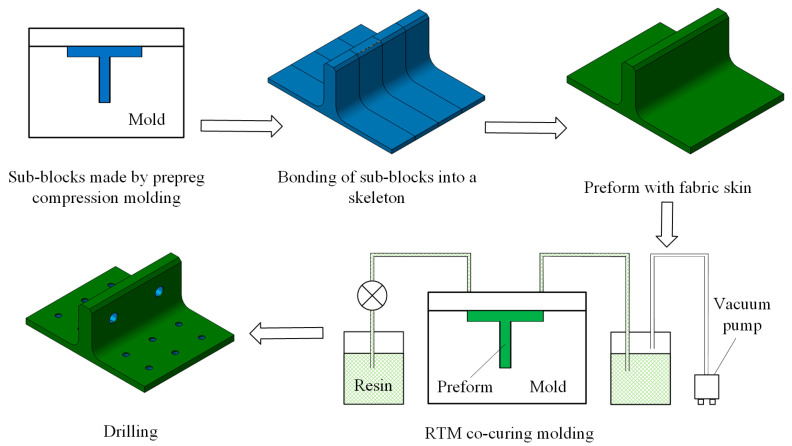
Manufacturing process of polymer composite bolted T-joint with novel configuration.

The geometric dimensions of the novel polymer composite bolted T-joint are depicted in Figure 3. The joint was fabricated using a novel prepreg-RTM co-curing molding process, with its configuration design shown in Figure 2. The internal load-carrying skeleton consists of 4 laminate sub-blocks, each 25 mm wide, all constructed using ZT7G/9368 unidirectional prepreg stacked along direction 3, as depicted in Figure 2. The stacking sequence for each sub-block is [0/+45/90/−45]_25s_, where the 0° direction corresponds to direction 1 in Figure 2, and the 90° direction corresponds to direction 2 in Figure 2. The external skin consists of a total of 5 layers, with 4 internal layers made of ZT7G warp-knitted fabric and the outermost ply made of ZT7G plain weave fabric. The thickness of each warp-knitted fabric layer is 0.125 mm, and the thickness of the plain weave fabric layer is 0.2 mm. The manufacturing process, as illustrated in Figure 4, involves 5 steps. Firstly, 4 laminate sub-blocks are prepared using prepreg compression molding and then they are bonded to form the internal skeleton. Subsequently, the dry warp-knitting fabric and plain weave fabric are wrapped around the skeleton to form the preform of the T-joint. Then, the preform is placed into a mold, and 6808 epoxy resin is injected for RTM co-curing molding. Finally, holes are drilled in the prepared T-joint. Therefore, the matrix material for the internal skeleton is primarily 9368 epoxy resin, while the matrix material for the external skin is mainly 6808 epoxy resin. The material properties of various types of fiber-reinforced polymer composite materials are presented in Table 1, while the material properties of 6068 epoxy resin are provided in Table 2. The materials and the nominal properties were all provided by Research Institute of Aerospace Special Materials and Processing Technology.

### 2.2. Test Procedure

As shown in Table 3, following the specimen design, three composite specimens with different stacking sequences of the skin were prepared, along with one specimen made of 2A12 aluminum alloy (which has significant potential applications in the aerospace industry [21,22]) of the same size, thus comparing the bending performance between the novel composite T-joint and the aluminum one. Here, the 0° direction corresponds to direction 3 in Figure 2, while the 90° direction is perpendicular to the 0° direction and normal to the outer surface of the T-joint.

**Figure 5:**
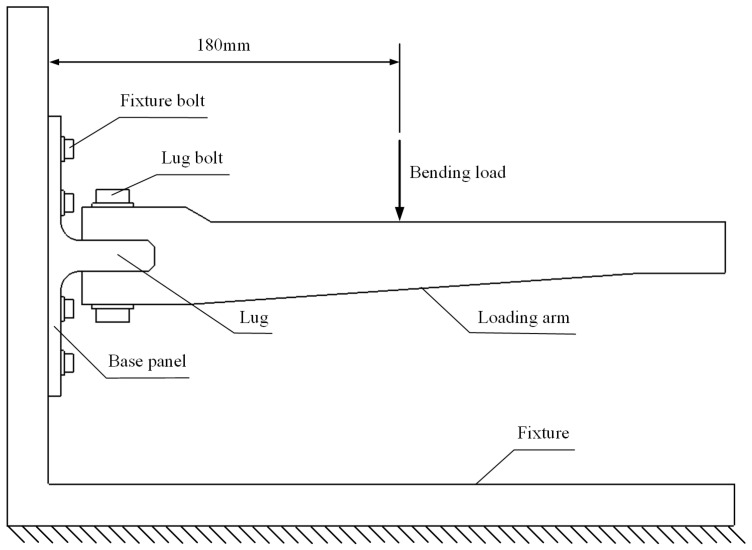
The diagram of loading scheme.

**Figure 6:**
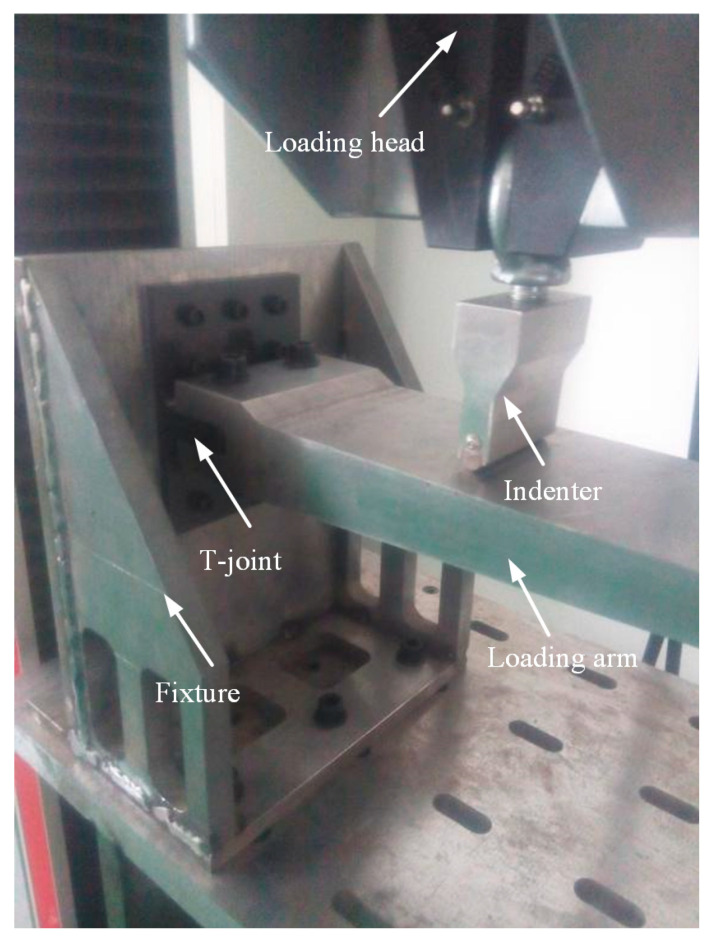
The experimental setup.

The loading scheme is depicted in Figure 5. The base panel is secured to the L-shaped fixture using twelve steel bolts with a diameter of 6 mm. The loading arm is connected to the lug using two steel bolts with a diameter of 10 mm. The distance between the loading point and the bottom surface of the joint is 180 mm. The experimental setup is illustrated in Figure 6. The L-shaped fixture is fixed on the test platform and reinforced with rib plates welded on both sides to provide sufficient bending stiffness. The load is applied downward by the indenter connected to the loading head of the INSTRON 8801 test machine (Norwood, MA, USA) with a speed of 0.5 mm/min, exerting bending load on the joint. The load and displacement of the indenter are recorded in real time by the sensors of the testing machine. The materials for the L-shaped fixture, loading arm, and indenter are all 45# steel. The properties of 45# steel are listed in Table 3.

### 2.3. Results and Discussion

**Figure 7:**
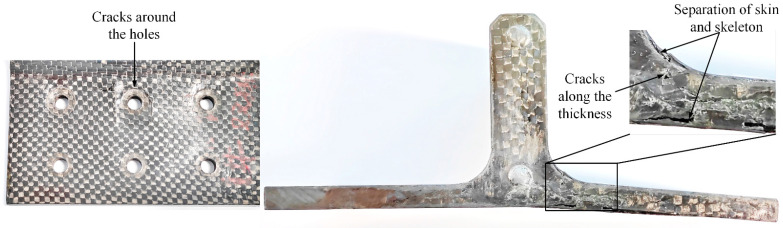
Damage morphologies of the specimen.

**Figure 8:**
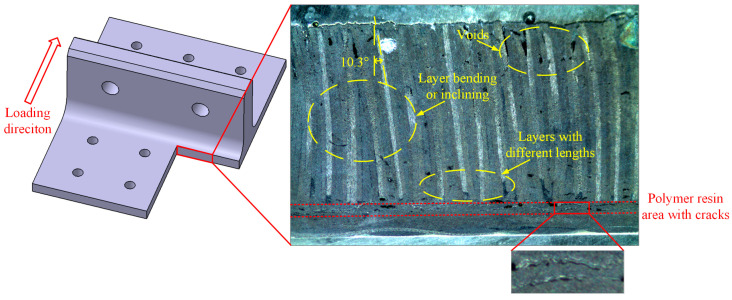
Profile of base panel near the R-corner area after the test.

**Figure 9:**
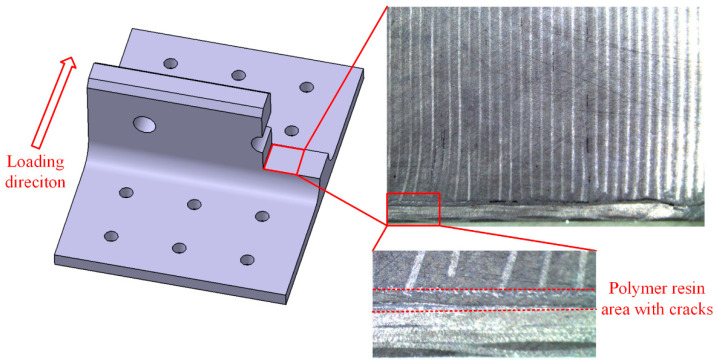
Profile of lug near the R-corner region after the test.

The post-test damage morphologies are depicted in Figure 7. The visible damage to the composite bolted T-joint includes cracks around the holes on the base panel, the separation of the skin and skeleton, and cracks along the thickness direction of the skeleton at the R-corner area. As shown in Figure 8 and Figure 9, the cross-sectional view of the R-corner area after the test reveals the presence of a polymer resin area with a non-negligible thickness between the skin and skeleton, with noticeable cracks within this resin area, while no significant delamination is observed in the skin. It can be inferred that the failure of the resin area between the skin and skeleton leads to their final separation. Additionally, defects such as voids, layer bending or inclining, and uneven ply lengths are present within the skeleton.

**Figure 10:**
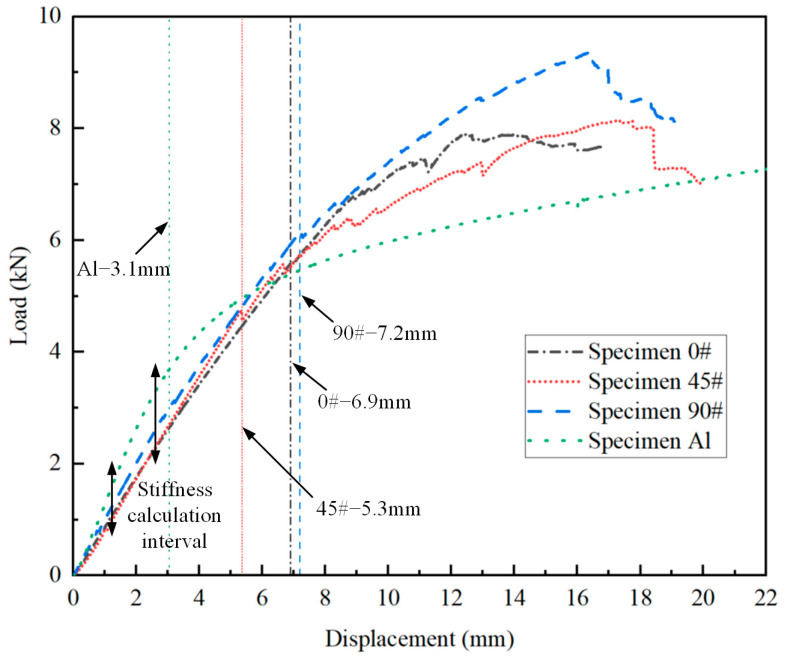
Load–displacement curves obtained from the tests.

The load–displacement curves obtained from the tests are presented in Figure 10, with the maximum displacement corresponding to significant load changes annotated on the curves. The bending stiffness of each joint is calculated using the linear segments of the data, and the load at which significant load changes occur is taken as the yield limit load for each joint. For the composite T-joints, the maximum load from the load–displacement curve is considered the ultimate load. The test results are summarized in Table 4. It is observed that before plastic deformation occurs in the aluminum T-joint, the bending stiffness of the composite T-joint is lower than that of the aluminum one, but the yield limit load of the composite T-joint is significantly higher than that of the aluminum one. Comparing the test results of specimen Al and Specimen 90#, the bending stiffness of the composite T-joint is 0.81 times that of the aluminum one, while the yield limit load is 1.65 times higher, with the weight being only 55.4% of the aluminum one. Clearly, the novel polymer composite bolted T-joint exhibits an excellent bending performance.

Comparing the test results of the three composite T-joints, an increasing trend is observed in the bending stiffness and ultimate load of the composite T-joint as the number of 90° layers in the skin increases and the number of 0° layers decreases. Furthermore, Specimen 90# exhibits the highest ultimate load and a smoother change in the load–displacement curve, mainly due to the relatively smaller influence of internal defects in this specimen. Thus, to obtain a finite element model with higher accuracy, it is advisable to establish the finite element model based on the parameters of Specimen 90#.

## 3. Numerical Study

### 3.1. Finite Element Model

Finite element models were established by using ABAQUS 2021 commercial software to analyze the stress distribution and corresponding failure mechanisms of the novel polymer composite bolted T-joint under a bending load. As mentioned earlier, there exists a layer of 6068 epoxy resin area between the skin and skeleton of the composite T-joint, with a nominal thickness of 0.3 mm. In addition, although the bonding interfaces between the composite materials and polymer resin adhesive play a significant role in the connection effectiveness and mechanical properties [23,24], as shown in Figure 8, the experimental results indicate that the failure of the resin area itself leads to the final separation of the skin and skeleton. Additionally, adding two layers of cohesive units between the skin, skeleton, and resin area would greatly increase the difficulty of the model convergence. Therefore, a layer of resin element was established between the skin and skeleton to represent the resin area and the bonding interfaces were assumed to be perfectly bonded.

Due to the large number of ply layers in the internal skeleton laminate, the precise modeling of each ply layer would significantly increase the computational costs and may lead to convergence issues. The Composite Layup function in ABAQUS was utilized to simplify the modeling of the multi-ply laminate by establishing multiple section points within a single element to represent the [0/+45/90/−45] periodic ply sequence. This method can balance the computational costs and accuracy, as demonstrated by Tserpes et al. [25]. Additionally, to optimize the computational efficiency, all the components of the T-joint and test fixtures were modeled using C3D8R elements, an eight-node linear brick element with reduced integration, known for its efficiency in large models.

**Figure 11:**
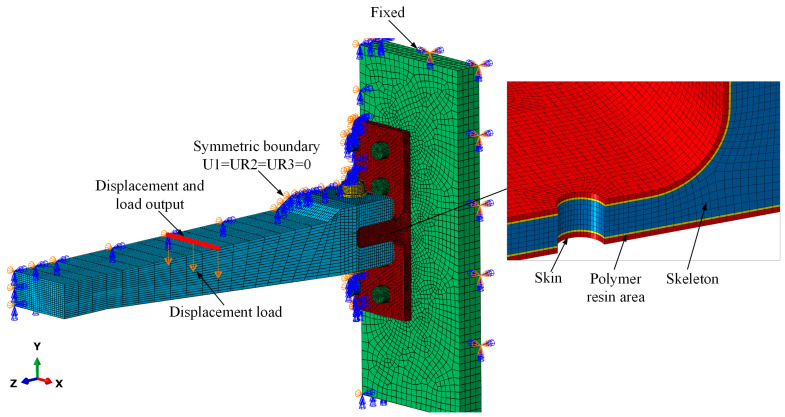
Boundary conditions, loading condition, and nodes for result output in FE model.

Figure 11 depicts the finite element model, labeled as the FE model, of the T-joint under a bending load with boundary conditions applied. The mesh near the bolt holes of the T-joint was optimized to better capture the concentrated stresses at the contact interfaces. Solid models of the loading arm, bolts, and fixture were established to accurately represent the loading conditions of the T-joint. The contact interactions between the solid entities were defined as surface-to-surface contacts with a friction coefficient of 0.2. Exploiting the geometric symmetry of the structure, only half of the model was constructed to reduce the computational costs, with necessary symmetry constraints applied to the symmetric plane to restrict out-of-plane displacements. Geometric nonlinear analysis was employed to account for relatively significant deformations, especially at the lug region of the T-joint.

Furthermore, a finite element model, labeled as the FE model (no resin), was created to validate the significance of the presence of the polymer resin area between the skin and skeleton. The FE model (no resin) maintained the same geometry as the FE model but the resin area was removed by proportionally increasing the thickness of the skin ply layers from 0.7 mm to 1 mm. The number and stacking sequence of the skin ply layers remained unchanged. All other settings in the FE model (no resin) remained consistent with the original model.

### 3.2. Material Damage Models

The accurate prediction of failure modes is a critical requirement for validating the accuracy of finite element models. Appropriate failure criteria are crucial for predicting failure modes and damage propagation process. To date, due to the occurrence of various failure behaviors in composite materials, numerous failure criteria tailored to different failure behaviors have been proposed. For unidirectional laminated composite plates, the 3D Hashin criteria can predict fiber and matrix failure behaviors [26,27,28], while the Ye delamination criteria can effectively predict interlaminar failure behavior [29,30,31], and the Chang fiber shear failure criteria can predict fiber matrix shear failure behavior [32]. Therefore, for the internal skeleton of the composite T-joints, a hybrid failure criterion based on the 3D Hashin criteria, Ye delamination criteria, and Chang fiber shear failure criteria was adopted.

According to the experimental results, significant separation occurred in the R-corner region between the skin and skeleton, but the separation interface mainly occurred in the polymer resin area between the skin and skeleton, with almost no delamination within the skin ply layers. Therefore, it is necessary to assess the failure behavior in the resin area while the delamination failure within the skin ply layers can be ignored. Since the plain weave fabric of the composite materials has the same fiber content in the warp and weft directions, the effect of matrix damage on the in-plane mechanical behavior can almost be neglected. Based on the analysis above, the Von Mises stress failure criterion is adopted to predict the failure behavior of the resin, which is suitable for analyzing the failure behavior of isotropic plastic materials. Simultaneously, only the fiber failure behavior of the plain weave fabric and the in-plane fiber and matrix failure behaviors of the warp-knitted fabric are considered, with the 3D Hashin criteria used as their failure criteria. Various failure modes and their failure criteria are listed as shown in Equations (1)–(8).

Fiber tensile failure (*σ*_11_ > 0):

(1) $${\left(\frac{\sigma_{11}}{X_{T}}\right)}^{2}+{\left(\frac{\tau_{12}}{S_{12}}\right)}^{2}+{\left(\frac{\tau_{13}}{S_{13}}\right)}^{2}\geq 1$$

Fiber compression failure (*σ*_11_ < 0):

(2) $${\left(\frac{\sigma_{11}}{X_{C}}\right)}^{2}\geq 1$$

Matrix tensile failure (*σ*_22_ > 0):

(3) $${\left(\frac{\sigma_{22}}{Y_{T}}\right)}^{2}+{\left(\frac{\tau_{12}}{S_{12}}\right)}^{2}+{\left(\frac{\tau_{23}}{S_{23}}\right)}^{2}\geq 1$$

Matrix compression failure (*σ*_22_ < 0):

(4) $${\left(\frac{\sigma_{22}}{Y_{C}}\right)}^{2}+{\left(\frac{\tau_{12}}{S_{12}}\right)}^{2}+{\left(\frac{\tau_{23}}{S_{23}}\right)}^{2}\geq 1$$

Fiber matrix shear failure (*σ*_11_ < 0):

(5) $${\left(\frac{\sigma_{11}}{X_{C}}\right)}^{2}+{\left(\frac{\tau_{12}}{S_{12}}\right)}^{2}+{\left(\frac{\tau_{13}}{S_{13}}\right)}^{2}\geq 1$$

Delamination in tension (*σ*_33_ ≥ 0):

(6) $${\left(\frac{\sigma_{33}}{Z_{T}}\right)}^{2}+{\left(\frac{\tau_{13}}{S_{13}}\right)}^{2}+{\left(\frac{\tau_{23}}{S_{23}}\right)}^{2}\geq 1$$

Delamination in compression (*σ*_33_ < 0):

(7) $${\left(\frac{\sigma_{33}}{Z_{C}}\right)}^{2}+{\left(\frac{\tau_{13}}{S_{13}}\right)}^{2}+{\left(\frac{\tau_{23}}{S_{23}}\right)}^{2}\geq 1$$

Polymer resin failure:

(8) $$\frac{\sigma_{\mathrm{mises}}}{\sigma_{T}}\geq 1$$

where *σ*_11_, *σ*_22_, and *σ*_33_ are the normal stress components along the *X*, *Y*, and *Z* directions, respectively, as shown in Figure 11. *τ*_12_, *τ*_13_, and *τ*_23_ are the shear stress components. *σ*_mises_ is the Mises stress. *X*_T_ and *X*_C_ are the tensile and compressive strengths along the longitudinal direction. *Y*_T_ and *Y*_C_ are the tensile and compressive strength along the transverse direction. *Z*_T_ and *Z*_C_ are the tensile and compressive strength along the thickness direction. *S*_12,_ *S*_13_, and *S*_23_ are the shear strengths.

**Figure 12:**
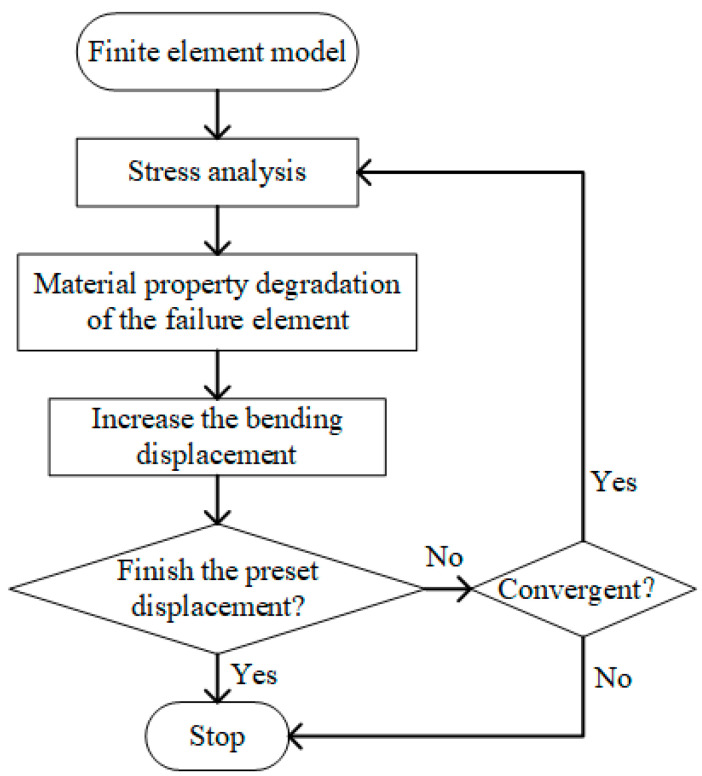
Flow chart of finite element model.

In finite element models, as soon as the failure is reached, the material properties of the failed elements need to be degraded based on the material property degradation rules [33]. To ensure the convergence of the model, the value of the material property after degradation should not be too small. In this investigation, the material property degradation rules for the composite bolted T-joint are shown in Table 5. Cheng et al. [14,15] utilized these material property degradation rules to numerically study the bending performance of a composite bolted π-joint and obtained highly accurate simulation results. The corresponding user subroutine USDFLD was developed to implement the determination of element failure and material property degradation. The computational process of the finite element model is illustrated in Figure 12.

### 3.3. Verification of the Model

**Figure 13:**
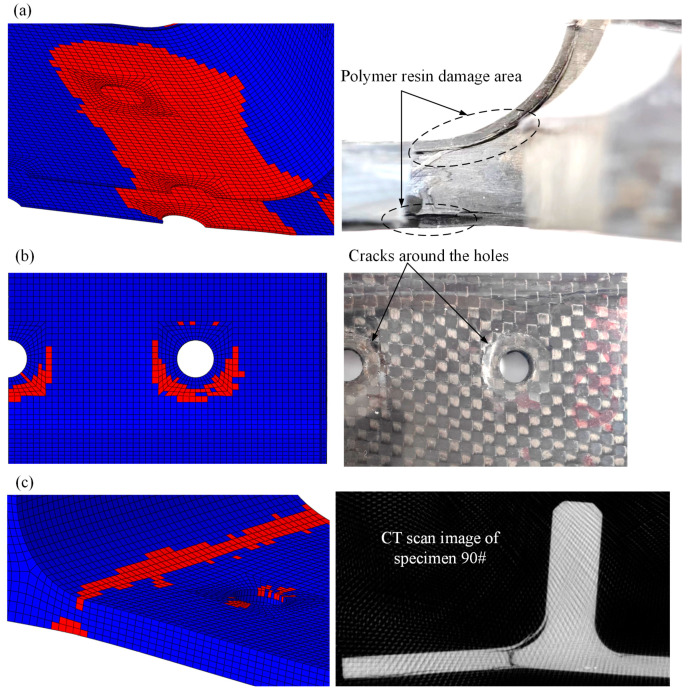
Comparison of numerical and experimental damage morphologies: polymer resin damage between skin and skeleton (**a**), skin damage area around the hole (**b**), and skeleton damage area along the thickness (**c**).

**Figure 14:**
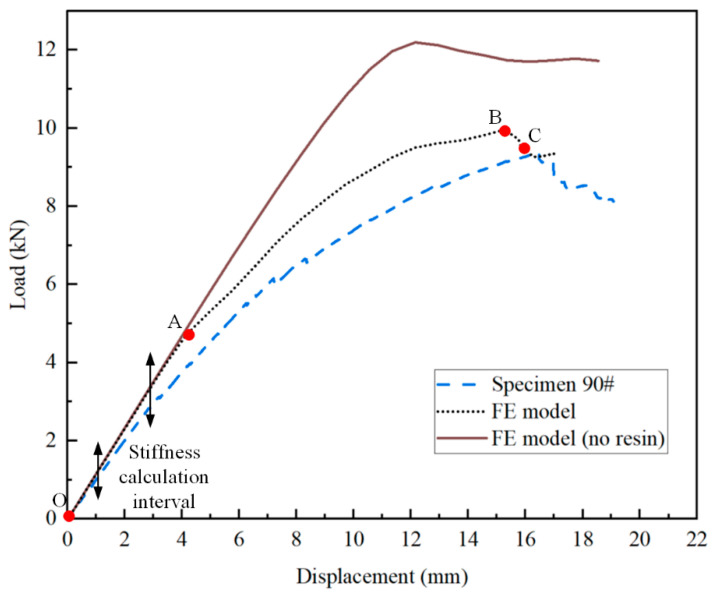
Load–displacement curves for specimen 90# by experimental and numerical analysis. (For Points O–C: O is the starting point, A is the damage initiation point, B is the ultimate load point and C is a point in the final destruction process).

As illustrated in Figure 7 and Figure 13, the damage morphology obtained from the experiments and finite element models is depicted. It is observed that the FE model successfully simulates the damage in the polymer resin area between the skin and skeleton, the damage near the holes on the surface, as well as the damage along the thickness direction at the R-corner area of the skeleton, which indicates a high level of consistency between the finite element and the experimental results. As shown in Figure 14 and Table 6, a comparison between the computational results of the FE model and experimental results is provided. It is noted that the variation trend of the load–displacement curve and the ultimate load obtained from the FE model closely align with the experimental results. The overestimation of the bending stiffness obtained from the FE model is primarily due to the neglect of various significant defects present in the composite T-joint, which lead to a decrease in its performance and subsequently reduce the bending stiffness of the joint.

Comparing the load–displacement curves obtained from the FE model and FE model (no resin), within the stiffness calculation interval, the two curves largely coincide, indicating that proportionally increasing the thickness of the skin layers in the FE model (no resin) has a minimal effect on the bending performance of the joint. However, as displacement increases, the load–displacement curve of the FE model (no resin) exhibits a significantly higher load, deviating notably from the experimental results. This indicates the substantial impact of the polymer resin area between the skin and skeleton on the bending performance of the composite T-joint, necessitating the incorporation of a layer of resin element in the finite element model.

In conclusion, the finite element model, considering the polymer resin area between the skin and skeleton, provides a good reflection of the bending loading process of the novel polymer composite bolted T-joints with a decent simulation accuracy.

### 3.4. Failure Mechanism

As depicted by the load–displacement curve obtained from the FE model in Figure 14, the damage propagation process of the polymer composite bolted T-joint can be divided into three stages. Based on the validated finite element model, the following findings are obtained.

**Figure 15:**
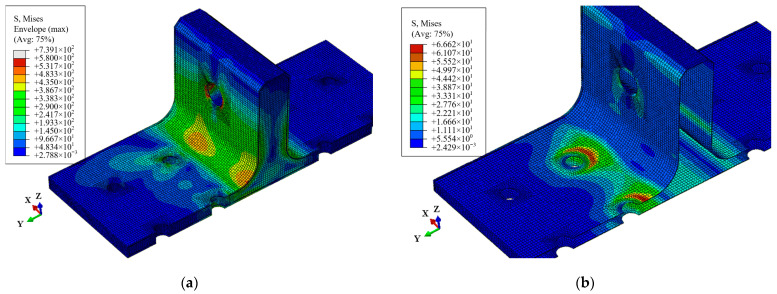
Mises stress distribution at point A: the whole T-joint (**a**) and polymer resin area (**b**).

**Figure 16:**
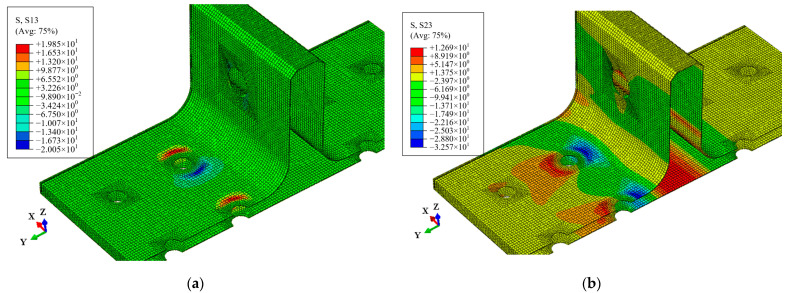
Shear stress distribution of the polymer resin area at point A: shear stress S13 (**a**) and shear stress S23 (**b**).

During the OA stage, there is no internal damage within the T-joint, and the load increases linearly with displacement. Upon reaching point A, the distribution of the Mises stress in the whole T-joint and polymer resin area is illustrated in Figure 15. Apart from the area near the holes on the lug, the T-joint experiences the maximum load at the R-corner area, while the resin area exhibits significant Mises stress near the holes on the base panel; thus, damage initially occurs within the resin near the holes before spreading to the skin and skeleton. Figure 16 shows the out-of-plane shear stress distribution in the polymer resin area at point A, indicating that significant out-of-plane shear stress is concentrated near the holes before the occurrence of damage, and this concentration leads to the initiation of damage. This also indicates that the primary cause of resin area failure is the shear force between the skin, skeleton, and resin area.

**Figure 17:**
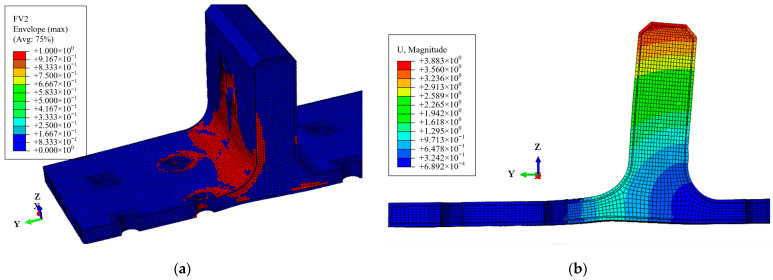
Damage distribution at point B (**a**). Deformation of T-joint at point B (**b**).

During the AB stage, the damage within the skin, skeleton, and resin area continuously expands from the areas near the holes towards the edges. Upon reaching point B, the damage and deformation of the composite bolted T-joint are illustrated in Figure 17, where the damage on the skin has extended to the edges, and the damage within the skeleton laminate has penetrated the entire base panel, which leads to the final failure of the joint. Simultaneously, noticeable deformation has occurred at the R-corner area of the T-joint. After point B, the R-corner area of the composite bolted T-joint loses its load-carrying capacity, and the bending load begins to decrease.

A further comparison between the curves obtained from the FE model and FE model (no resin) in Figure 14 reveals that when there is no polymer resin area between the skin and skeleton, the load at the occurrence of damage is higher, and the ultimate load of the joint is also higher. This demonstrates that the presence of the resin area leads to a decrease in the bending performance of novel polymer composite bolted T-joints.

## 4. Influencing Factors Analysis

As previously discussed, since the novel composite bolted T-joint primarily relies on the skeleton laminate for load carrying, variations in the stacking sequence of the skeleton may have an impact on the bending performance of the joint. In addition, the experimental results indicate the presence of defects such as layer inclining within the skeleton, which can affect the bending performance of the joint. Furthermore, the experimental results also suggest that the stacking sequence of the skin influences the bending performance of the joint. Therefore, based on the validated finite element model, numerical investigations can be conducted to study the effects of variations in the stacking sequence and layer inclining within the skeleton, as well as the stacking sequence of the skin, on the bending performance of the composite bolted T-joint.

### 4.1. Effect of the Stacking Sequence of the Skeleton

**Figure 18:**
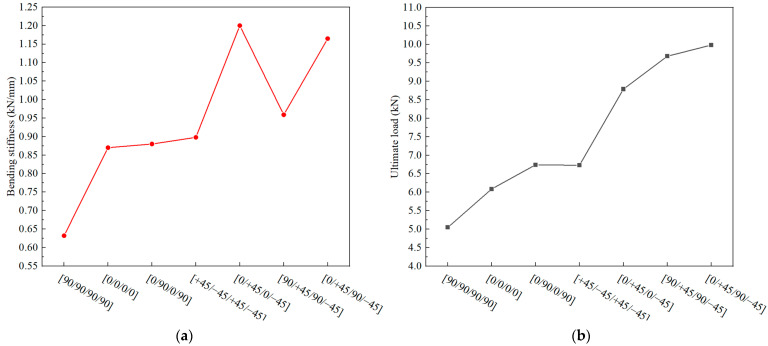
Variation in mechanical properties with the stacking sequence of the skeleton: bending stiffness (**a**) and ultimate load (**b**).

Compared to a composite T-joint with a conventional configuration, the novel composite bolted T-joint can convert interlaminar loads of the skeleton into in-plane loads, thereby enhancing the overall load-carrying capacity of the joint. With the increase in the load-carrying capacity, the novel-configuration T-joint also introduces the parameter of the stacking sequence of the skeleton as a performance-influencing factor. Investigating the effect of the stacking sequence on the bending performance of the novel-configuration T-joint can guide designers in selecting a more rational stacking sequence for the skeleton. Finite element models were established with stacking sequences of [0/0/0/0], [0/+45/0/−45], [0/90/0/90], [+45/−45/+45/−45], [90/+45/90/−45], and [90/90/90/90], in addition to the [0/+45/90/−45] stacking sequence. The computational results are depicted in Figure 18.

The computational results indicate that when the skeleton lacks ±45° layers, the bending performance of the T-joint significantly decreases, primarily because ±45° layers can enhance the shear strength of the joint near the bolt holes. Moreover, when the skeleton contains ±45° layers but lacks 0° or 90° layers, the bending performance of the T-joint also declines to some extent, especially when only ±45° layers are present in the skeleton, leading to a significant decrease in the bending performance. Hence, when the T-joint primarily experiences bending loads, the optimal stacking sequence for the skeleton is [0/+45/90/−45]_ns_.

### 4.2. Effect of the Layer Inclining of the Skeleton

**Figure 19:**
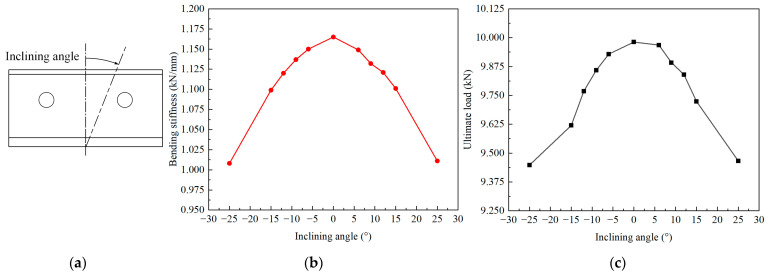
Diagram of inclining angle (**a**). Variation in mechanical properties with the change in the inclining angle of the layer in the skeleton: bending stiffness (**b**) and ultimate load (**c**).

The skeleton is a critical component responsible for bearing loads in the novel composite bolted T-joint. Investigating the effect of layer inclining within the skeleton on the bending performance can provide an acceptable range for layer inclining defects when manufacturing the novel T-joints. Considering the symmetry of the finite element model, the case where layers are symmetrically inclined about the T-joint’s symmetry plane can be studied. As seen in Figure 8, the angle of layer inclining is approximately around 10°. Therefore, while keeping other parameters constant, only changing the angle of the layer incline to angles of 6°, 9°, 12°, 15°, and 25°, the numerical calculation results shown in Figure 19 are obtained.

It can be observed that when layers are symmetrically inclined about the symmetry plane, both the bending stiffness and ultimate load decrease nonlinearly with increasing inclining angle. Additionally, the bending stiffness and ultimate load under the same absolute value of the negative and positive inclining angle are essentially the same. Comparing the bending stiffness and ultimate load under layer inclining with the scenario without layer inclining, when the absolute value of the inclining angle is less than 12°, the decrease in the bending stiffness is no more than 2%, and the decrease in the ultimate load is no more than 5%. Therefore, if the criterion is set that the decrease in the bending stiffness and ultimate load should not exceed 5%, it can be considered that symmetric layer inclining within the novel T-joint has a minimal impact on its bending performance when the absolute value of the inclining angle is less than 12°.

### 4.3. Effect of the Stacking Sequence of the Skin

**Figure 20:**
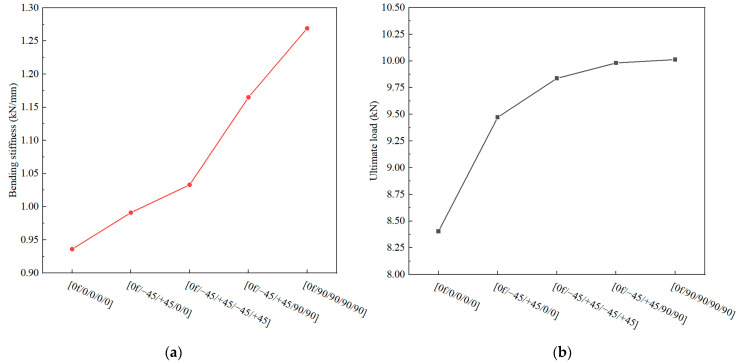
Variation in mechanical properties with changes in the stacking sequence of the skin: bending stiffness (**a**) and ultimate load (**b**).

According to the experimental results shown in Figure 10, it is evident that the stacking sequence of the skin affects the bending stiffness and ultimate load of the novel T-joint. However, due to various defects within the test specimens, significant fluctuations in the bending load occur before reaching the ultimate load, which significantly impacts the determination of the ultimate load. More accurate comparative data on the influence of the skin stacking sequence on the bending performance of the T-joint can be obtained using the validated finite element model. In addition to the stacking sequence used in the specimens, two additional stacking sequences, namely, [0_f_/0/0/0/0] and [0_f_/90/90/90/90], were included in the finite element models. All the models maintain consistent settings except for the stacking sequence. The numerical calculation results are shown in Figure 20.

It is observed that as the proportion of 90° layers increases, both the bending stiffness and ultimate load of the T-joint increase. Conversely, an increase in the proportion of 0° layers leads to a decrease in the bending stiffness and ultimate load. The effect of the 45° layers lies between that of the 90° layers and 0° layers. Furthermore, increasing the proportion of 90° layers has a more significant impact on the increase in the bending stiffness compared to the increase in the ultimate load. While the influence of the 45° layers and 90° layers on the ultimate load is nearly similar, the effect of the 90° layers on the bending stiffness is more pronounced. Therefore, when designing the stacking sequence of the skin for the novel composite bolted T-joint, in addition to considering factors such as wear resistance, increasing the proportion of 90° layers can enhance the bending stiffness and ultimate load of the joint.

## 5. Conclusions

A set of polymer composite bolted T-joints with a novel configuration consisting of an internal skeleton and external skin was fabricated using a prepreg-RTM co-curing molding process. The bending performance of these novel T-joints was experimentally investigated. A finite element model with a polymer resin area between the skin and skeleton was established, and verified by the experimental results. The validated finite element model was used to analyze the damage propagation process and failure mechanism of the novel T-joint. Finally, numerical studies were conducted on three performance-influencing factors related to the layer characteristics of the skin and skeleton. The following conclusions can be drawn:

(1) The bending stiffness and the yield limit load of the novel composite T-joint are 0.81 times and 1.65 times that of the 2A12 aluminum T-joint, respectively, while at only 55.4% of its weight.
(2) There exists a layer of polymer resin area between the skin and skeleton of the novel T-joint, and the presence of this area can lead to a significant decrease in the bending performance of the novel T-joint.
(3) The finite element model with a layer of resin area between the skin and skeleton exhibits excellent accuracy, and the damage of the novel T-joint initiates in the resin area near the bolt holes on the base panel. Subsequently, the damage within the skin, skeleton, and resin area expands from the areas near the holes towards the edges. Finally, damage within the skeleton penetrates the entire base panel, which results in the final failure of the novel T-joint.
(4) When primarily subjected to bending loads, the optimal stacking sequence for the skeleton is [0/+45/90/−45]_ns_. Furthermore, when the symmetrically inclining angle of the layer in the skeleton is less than 12°, the decrease in the bending stiffness and in the ultimate load are no more than 2% and 5%, respectively.
(5) For the stacking sequence of the skin, the more 90° layers there are, the better the bending performance, whereas the more 0° layers there are, the poorer the bending performance. The influence of the 45° layers lies between that of the 90° layers and 0° layers.

## Figures and Tables

**Table 1:** Material properties of polymer composite bolted T-joint.

Item	ZT7G/9368	ZT7G Warp-Knitting Fabric/6808	ZT7G Plain Weave Fabric/6808
Longitudinal elastic modulus, *E*_11_ (GPa)	132	132	61.5
Transverse elastic modulus, *E*_22_ (GPa)	8.55	9	61.5
Through-thickness elastic modulus, *E*_33_ (GPa)	8.55	9	8
In-plane shear modulus, *G*_12_ (GPa)	4.32	5.9	4.89
Out-of-plane shear modulus, *G*_13_ (GPa)	4.32	5.9	4.89
Out-of-plane shear modulus, *G*_23_ (GPa)	3.35	3.3	4.89
Poisson’s ratio, *μ*_12_	0.33	0.3	0.06
Poisson’s ratio, *μ*_13_	0.33	0.3	0.3
Poisson’s ratio, *μ*_23_	0.33	0.3	0.3
Longitudinal tensile strength, *X*_T_ (MPa)	2200	2170	842
Longitudinal compressive strength, *X*_C_ (MPa)	1200	938	628
Transverse tensile strength, *Y*_T_ (MPa)	38	36.2	842
Transverse compressive strength, *Y*_C_ (MPa)	196	192	628
Through-thickness tensile strength, *Z*_T_ (MPa)	38	36.2	-
Through-thickness compressive strength, *Z*_C_ (MPa)	196	192	-
In-plane shear strength, *S*_12_ (MPa)	90	88.3	92.2
Out-of-plane shear strength, *S*_13_ (MPa)	90	88.3	-
Out-of-plane shear strength, *S*_23_ (MPa)	86	68.3	-

**Table 2:** Material properties of epoxy 6808 and 45# steel.

Material	Modulus, *E* (GPa)	Poisson’s Ratio, *μ*	Strength, *σ*_T_ (MPa)
Epoxy resin 6808	3.3	0.3	65.6
45# steel	210	0.33	-

**Table 3:** Arrangement of test specimens.

Specimen	Number of Specimens	Stacking Sequence of the Skin	Weight (g)
Specimen 0#	1	[0_f_/−45/+45/0/0]	226.22
Specimen 45#	1	[0_f_/−45/+45/−45/+45]	226.14
Specimen 90#	1	[0_f_/−45/+45/90/90]	227.35
Specimen Al	1	-	410.51

**Table 4:** Test results.

Specimen	Bending Stiffness (kN/mm)	Yield Limit Load (kN)	Ultimate Load (kN)
Specimen 0#	0.873	5.584	7.882
Specimen 45#	0.953	4.702	8.118
Specimen 90#	0.992	6.093	9.360
Specimen Al	1.221	3.694	-

**Table 5:** Material property degradation rules.

Material	Failure Mode	Degradation Rule
ZT7G/9368	Fiber breakage	*E*_11_ = 0.1*E*_11_
	Matrix cracks	*E*_22_ = 0.2*E*_22_, *G*_12_ = 0.2*G*_12_, *G*_23_ = 0.2*G*_23_
	Delamination	*E*_33_ = 0.01*E*_33_, *G*_13_ = 0.01*G*_13_, *G*_23_ = 0.01*G*_23_, *μ*_13_ = 0.01*μ*_13_, *μ*_23_ = 0.01*μ*_23_
	Fiber–matrix shear failure	*G*_12_ = 0.2*G*_12_, *μ*_12_ = 0.2*μ*_12_
ZT7G warp-knitting fabric/6808	Fiber breakage	*E*_11_ = 0.1*E*_11_
	Matrix cracks	*E*_22_ = 0.2*E*_22_, *G*_12_ = 0.2*G*_12_, *G*_23_ = 0.2*G*_23_
ZT7G plain weave fabric/6808	Warp fiber breakage	*E*_11_ = 0.1*E*_11_, *G*_12_ = 0.2*G*_12_, *G*_13_ = 0.01*G*_13_, *μ*_12_ = 0.01*μ*_12_, *μ*_13_ = 0.01*μ*_13_
	Weft fiber breakage	*E*_22_ = 0.1*E*_22_, *G*_12_ = 0.2*G*_12_, *G*_23_ = 0.01*G*_23_, *μ*_12_ = 0.01*μ*_12_, *μ*_23_ = 0.01*μ*_23_
Epoxy resin 6808	Polymer resin failure	*E* = 0.03*E*, *μ* = 0.03*μ*

**Table 6:** Comparison of experimental and numerical results.

	Bending Stiffness (kN/mm)	Ultimate Load (kN)
Specimen 90#	0.992	9.360
FE model	1.165	9.981
Difference (%)	17.4	6.63

## Data Availability

Data are contained within the article.
